# OnabotulinumtoxinA Reduces Temporal Pain Processing at Spinal Level in Patients with Lower Limb Spasticity

**DOI:** 10.3390/toxins11060359

**Published:** 2019-06-20

**Authors:** Roberto De Icco, Armando Perrotta, Eliana Berra, Marta Allena, Enrico Alfonsi, Stefano Tamburin, Mariano Serrao, Giorgio Sandrini, Cristina Tassorelli

**Affiliations:** 1 Neurorehabilitation Unit, IRCCS Mondino Foundation, 27100 Pavia, Italy; eliana.berra@mondino.it (E.B.); marta.allena@mondino.it (M.A.); giorgio.sandrini@unipv.it (G.S.); cristina.tassorelli@unipv.it (C.T.); 2 Department of Brain and Behavioral Sciences, University of Pavia, 27100 Pavia, Italy; 3 IRCCS Neuromed, 86077 Pozzilli, IS, Italy; arm.perrotta@gmail.com; 4 Department of Neurophysiopathology, IRCCS Mondino Foundation, 27100 Pavia, Italy; enrico.alfonsi@mondino.it; 5 Department of Neurosciences, Biomedicine and Movement Sciences, University of Verona, 37134 Verona, Italy; stefano.tamburin@univr.it; 6 Department of Medical and Surgical Sciences and Biotechnologies, Sapienza University of Rome, 00100 Rome, Italy; mariano.serrao@uniroma1.it

**Keywords:** botulinum toxin, spasticity, neuropathic pain, lower limb reflex, spinal temporal summation, stroke, multiple sclerosis, spinal cord injury

## Abstract

Spasticity is a muscle tone disorder associated with different neurological conditions. Spasticity could be associated with pain, high disability, poor functional recovery, and reduced quality of life. Botulinum neurotoxin type A (BoNT-A) is considered a first-line treatment for spasticity and, more recently, it also represents a therapeutic option for various chronic pain conditions. In this open label study, we aim to evaluate the effect of the BoNT-A on the spinal nociception in patients affected by spasticity of the lower limbs with associated pain with predominantly neuropathic features. Ten patients with stroke, 10 with multiple sclerosis and 5 with spinal cord injury were enrolled in the study. They were tested with clinical scales (neuropathic pain scale inventory (NPSI), numerical rating scale (NRS), modified Ashworth scale (MAS) and with the nociceptive withdrawal reflex at lower limbs to explore the spinal temporal summation threshold at baseline and 30 day after BoNT-A injection. OnabotulinumtoxinA (50 to 200 units per site) was injected in the lower limb muscles according to the distribution of spasticity. No significant differences were found at baseline for neurophysiological features across groups. After the BoNT-A injection, we recorded a significant reduction in MAS and NRS scores. Regarding the neurophysiological parameters, we described a significant increase in the temporal summation threshold after the BoNT-A injection. Our data supports the hypothesis that peripherally injected OnabotulinumtoxinA modulates the excitability of spinal cord nociceptive pathways. This activity may take place irrespective of the effect of the drug on spasticity.

## 1. Introduction

Spasticity is a motor disorder characterized by a velocity dependent increase in muscle tone, with exaggerated tendon jerks, resulting from hyper-excitability of the stretch reflex, and is one component of the upper motor neuron syndrome [1]. There are several disorders in which spasticity occurs, including, among others, stroke, multiple sclerosis (MS) and spinal cord disease. Spasticity frequently develops in chronic stroke, where it is found in 38% of patients six months after the acute event [2] and its prevalence peaks at 73% in MS patients [3]. Spasticity represents a highly disabling neurological condition, in that it limits the functional recovery, and has a severe impact on the quality of life [4,5,6]).

Up to 65% of patients who develop spasticity also present painful symptomatology [7]. Pain management strategies, including physical and occupational therapies, as well as pharmacological and surgical interventions should be considered as part of spasticity treatment [8,9]. However, the relationship between spasticity and pain is poorly explored. In patients suffering from spasticity, pain has frequently nociceptive features, but up to 14% of subjects describe pain of neuropathic origin [7], suggesting the involvement of central mechanisms.

Botulinum neurotoxin type A (BoNT-A) is considered a safe and effective treatment for spasticity [6] and more recently it emerged also as an effective treatment for chronic pain conditions not associated with spasticity, such as migraine [10,11,12] and various types of neuropathic pain, including postherpetic neuralgia, trigeminal neuralgia, and neuropathic pain secondary to peripheral nerve and spinal cord lesions [13,14]. Indeed, although BoNT-A primarily causes chemodenervation of the neuromuscular junction, it may also modify the sensory feedback loop, possibly acting at the spinal cord level via axonal transport to induce central antinociceptive activity [15]. The proposed central mechanisms responsible for the antinociceptive activity of the BoNT-A include the inhibition of the release of pain modulators, such as substance P [16] and glutamate [17], as well as the excitability reduction of the wide dynamic range (WDR) neurons located in the dorsal horns of the spinal cord [18], probably via glutamate release reduction. In both animal models and humans, the excitability of the WDR neurons is supposed to be pivotal in physiological (discrimination) and pathological (central sensitization) nociception and their temporary frequency-dependent facilitation is referred to as temporal summation of pain [19].

In a recent study, we demonstrated that, in subjects with upper limb spasticity, the BoNT-A injection reduced in parallel the degree of spasticity and the size of the nociceptive withdrawal reflex (NWR) response of the upper limb, irrespective of the site of injection [20]. The NWR is an objective nociceptive reflex response mediated by a central circuitry sustained by WDR neurons [21], so these data support an intrinsic antinociceptive effect of the BoNT-A, encouraging further studies. In humans, the temporal summation threshold (TST) of the NWR of the lower limbs develops in parallel with the temporal summation of pain [21], which in turn directly reflects the excitability of the WDR neurons at spinal level [22,23]. Then, the study of the effects of the BoNT-A on the TST of the NWR in the lower limbs could improve our knowledge on the central antinociceptive mechanisms of BoNT-A taking place in the spinal cord.

The aim of the study was to evaluate the effect of BoNT-A on the spinal nociception in patients affected by spasticity of the lower limbs who reported pain with predominantly neuropathic features [24]. To this end, we enrolled patients with spasticity of various etiology (i.e., stroke, MS and spinal cord injury, SCI), and explored the TST of the NWR of the lower limb before and 30 days after the injection of BoNT-A.

## 2. Results

Ten patients with stroke (6 males, age: 66.5 ± 8.1, National Institute of Health Stroke Scale—NIHSS: 8.6 ± 4.0), 10 with MS (6 males, age: 49.9 ± 8.8, Expanded Disability Status Scale—EDSS: 7.3 ± 2.1) and 5 with SCI (5 males, 39.5 ± 9.2, American Spinal Injury Association Impairment Scale - ASIA: D) were studied. All clinical data are reported in Table 1. We found a significant difference in age and disease duration across groups at T0 because of younger age in the MS and SCI groups. Subjects with MS had lower Barthel Index and stroke patients had lower MAS score at the knee. The other demographical and clinical features considered for the statistical analysis, as well as previous exposure to BoNT-A and scores for disability and pain were comparable at baseline across groups. Fifty-two percent of patients received an OnabotulinumtoxinA injection for the first time.

**Figure 1:**
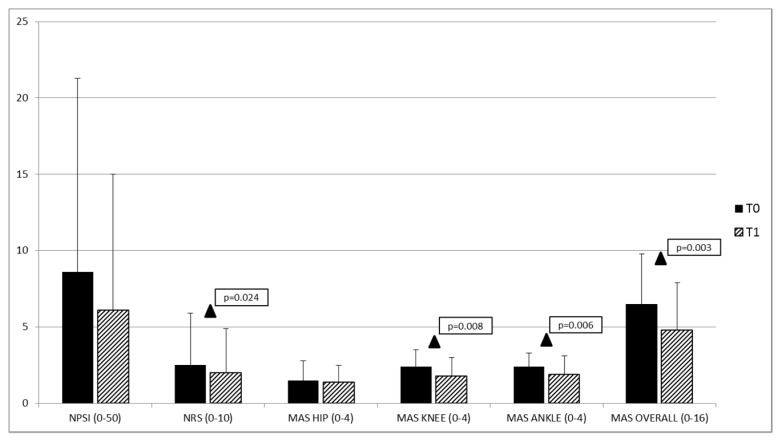
Mean values of the clinical parameters (NPSI, NRS and MAS) of the injected lower limb in the overall population at baseline (T0) and 30 days after the OnabotulinumtoxinA injection (T1). NPSI = neuropathic pain scale inventory; NRS = numerical rating scale; MAS = modified Ashworth scale. ▲ T0 vs. T1: *p* < 0.05.

When the clinical and psychophysical outcomes were considered, a significant reduction in modified Ashworth scale (MAS) (knee, ankle and overall) and numerical rating scale (NRS) (referred to the overall pain sensation) mean values was found in the patients overall considered for T1 vs. T0 comparison (Table 2 and Figure 1). We also observed a close-to-significant reduction in the neuropathic pain scale inventory (NPSI) score at T1 compared to T0 (Table 2 and Figure 1). For the other clinical outcomes, no significant difference was found between T0 and T1 (Table 2), either in the overall population or in single patient groups.

**Figure 2:**
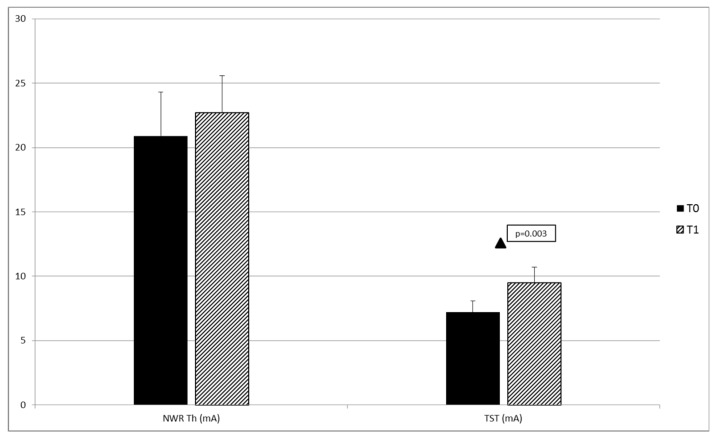
Mean values of the neurophysiological parameters of the injected lower limb in the overall population at baseline (T0) and 30 days after the OnabotulinumtoxinA injection (T1). NWR Th = nociceptive withdrawal reflex threshold; TST = temporal summation threshold of the NWR. ▲ T0 vs. T1: *p* < 0.05.

For neurophysiological outcomes, no significant differences were found at T0 between patient groups except for area (Table 3). A significant increase in NWR TST (primary outcome) was found in the overall population when comparing T1 to T0 (Table 4 and Figure 2). No significant differences in neurophysiological outcomes were found comparing T1 and T0 in single patient groups.

No significant correlation was found between neurophysiological and clinical variables, either in the overall population or in single patient groups.

## 3. Discussion

The results of the present study can be summarized as follows: OnabotulinumtoxinA injection (1) significantly increased the NWR TST in patients with spasticity and pain with predominantly neuropathic features; (2) significantly reduced the overall pain perception and; (3) significantly improved the MAS scores in the lower limb at T1. In parallel, we observed a nearly significant reduction in neuropathic severity pain score after OnabotulinumtoxinA injection.

As expected, our data confirmed the efficacy of OnabotulinumtoxinA in reducing spasticity and related overall pain sensation in a wide range of pathological conditions, including stroke, SM and SCI [14]. The new finding of this report is the concomitant significant reduction in temporal processing of nociceptive stimuli at the spinal level after OnabotulinumtoxinA injection, as documented by the increase in the TST of the NWR. From a neurophysiological point of view, the increase in the TST of the NWR represents an objective reduction in excitability of the nociceptive neurons at spinal level. NWR is a reliable measure of spinal nociception [21] and the temporal summation of the NWR represents a progressive increase in the size of the NWR response after a series of constant-intensity electrical stimuli activating A delta and C fibers. Temporal summation of the sensory stimuli is a form of neural plasticity related to the WDR neurons activity after constant-intensity stimulation of C fibers at ≥0.3 Hz. WDR neurons generate and amplify pain sensation via temporal integration of non-nociceptive and nociceptive afferents in a phenomenon referred to as temporal summation of pain [22,25], which is the human counterpart of the wind-up documented in animal models [22,23]. This feature of the WDR neurons is pivotal in physiological nociception for the discriminative analysis of pain sensation [26], as well as in pain chronification for the induction and maintenance of central sensitization [23,27,28,29,30].

The observed increase in TST of NWR supports the hypothesis that peripherally injected OnabotulinumtoxinA decreases central nervous signaling, thus reducing the WDR neuron activity [18] and the pain mediators release [16,17] at the spinal level. From a clinical point of view, the TST of the NWR reflects the shift of the sensory information from tactile to nociceptive and it is considered an affordable and objective representation of the temporal processing of nociceptive signals into the spinal cord [21,26,31] in both physiological and pathological conditions [32,33,34]. We may hypothesize that, while the significant reduction of the overall pain sensation could reflect the reduction in nociceptive pain due to the improvement of the spasticity, the trend toward a reduction in neuropathic pain components is in line with the reduction of the sensitization of the spinal nociceptive neurons.

Indeed, the reduction of the NPSI scores, even if it did not reach the statistical significance, might represent the clinical subjective counterpart of the reduction of the sensitization of the spinal nociceptive neurons. We speculate that the lack of statistical significance in NPSI scores could be related mostly to the sample size and the clinical heterogeneity of patients.

Regarding the relationship between OnabotulinumtoxinA injection, pain and spasticity, we are aware that the reduction of the spasticity can influence the anti-nociceptive effect of the OnabotulinumtoxinA on the NWR. However, recent evidences suggest that central post-stroke pain relief and spasticity improvement after OnabotulinumtoxinA injection were independent from each other, and with a different and typical temporal profile [35,36].

Only a few studies have investigated the effect of the botulinum toxin administration on the nociceptive reflex responses in animals, as well as in humans.

In animals, Favre-Guilmard et al. [37] demonstrated that unilateral OnabotulinumtoxinA administration increases the nociceptive withdrawal reflex threshold in rats and reduces hyperalgesia in experimental inflammatory models without affecting significantly the motor performances of the rats. Interestingly, a similar analgesic effect was observed in the contralateral paw, suggesting that the antinociceptive effects of the OnabotulinumtoxinA is unrelated to muscular relaxation and involve several pain processes.

Similarly, we recently demonstrated [20] that in subjects with subacute stroke, the injection of OnabotulinumtoxinA induces a diffuse significant reduction of the NWR-related responses also in non-injected muscles of the affected injected limb. Then, also in humans the anti-nociceptive effect of the OnabotulinumtoxinA seems to be independent of the effects on muscular relaxation and involving a global inhibitory effect on the cervical spinal neurons.

However, as the presence of muscle relaxation can overestimate the anti-nociceptive effect of the OnabotulinumtoxinA on the NWR. In the present study, to better understand the antinociceptive effect of the OnabotulinumtoxinA and to minimize the effect of the muscle relaxation in NWR responses, we studied the effect of the OnabotulinumtoxinA on the TST of the NWR, as it represents an objective evaluation of the functional activity of nociceptive spinal neurons. In addition, we avoided injecting the muscles directly involved in the reflex response such as the biceps femoris or adjacent thigh flexor muscles and we injected more distal muscles of the leg.

Our results showed that NWR area was not significantly reduced between T0 and T1 as well as the NWR Th was not significantly increased, demonstrating that the spasticity improvement per se was not able to significantly influence the nociceptive response. In parallel, the significant increase of the TST of the NWR after the OnabotulinumtoxinA injection indicated a significant inhibitory effect on sensory/nociceptive spinal neurons.

Our data show that OnabotulinumtoxinA injection improved clinical and neurophysiological outcomes in patients affected by neurological disorders causing spasticity and pain with neuropathic characteristics. We found no correlation between the degree of spasticity and the neurophysiological parameters at T1, which suggests that OnabotulinumtoxinA may have a direct effect on the excitability of spinal cord nociceptive pathways, irrespective of the effect on spasticity.

### Limitations of the study

Limitations of the present study included the small sample size, the open label design of the study and the heterogeneity of the patients that suggest caution in the interpretation of the present findings.

The enrollment of patients who were not taking drugs for spasticity and pain has probably contributed to select subjects with a relatively low NPSI and NRS scores, which limits the generalizability of our findings.

## 4. Conclusions

Our results encourage further studies to better characterize the modulation of the central nociceptive pathways induced by OnabotulinumtoxinA injection, to further support its role as a pharmacological treatment of pain with neuropathic component [38,39]. Moreover, they could help optimizing the OnabotulinumtoxinA protocols to achieve better and long-lasting results in the treatment of spasticity and associated pain.

## 5. Materials and Methods

The study was approved by the IRCCS Mondino Foundation Ethics Committee (CE n. 07/2013, Pavia, Italy, approved on July 2013) and was carried out following the guidelines for proper human research conduct in accordance with the Helsinki Declaration of the World Medical Association and its revisions. All the participants gave their written consent and were informed that they could withdraw from the experiments at any time.

### 5.1. Study Population

Patients with previous stroke, either ischemic or hemorrhagic, MS and SCI with evidence of spasticity and pain with neuropathic features (i.e., burning, electric-like, painfully cold, etc.) were recruited at Neurorehabilitation Unit of C. Mondino National Neurological Institute, Pavia, Italy.

The inclusion criteria were: (a) Male or female patients aged between 20 and 75 years; (b) onset of spasticity at least 6 months before; (c) spasticity in one or both lower limbs with a modified Ashworth scale (MAS) score of at least 2 in at least one lower limb; (d) residual motor function in the lower limb muscles (i.e., Medical Research Council scale score > 2/5); (e) last BoNT-A injection at least 6 months before.

The exclusion criteria were: (a) Severe osteoarticular degenerative or inflammatory disease in the lower limbs, myopathy or polyneuropathy that could hinder NWR acquisition; (b) contraindications to the use of BoNT-A (e.g., hypersensitivity to BoNT-A or any of the excipients, infection in the proposed site of injection, atrophy of muscle to inject); (c) psychiatric disorders; (d) Beck depression inventory (BDI) score > 19/63 (e) moderate to severe cognitive impairment (i.e., mini mental state examination, MMSE < 21/30); (f) inability to communicate with investigators or to answer the questionnaire.

No change in the treatment schedule was allowed from T0 to T1. No drugs or other interventions for neuropathic pain and spasticity [8,9,40] were allowed during the study. Previous exposure to medications for pain or spasticity did not represent an exclusion criterion, provided that before enrollment the subject underwent a wash-out period of one month in the case of drugs with a short half-life, or of at least five half-lives in the case of drugs with a long half-life. Previous exposure to BoNT-A injection was allowed, but a mandatory six-month wash-out period was required before enrollment.

### 5.2. Experimental Procedure

At enrollment the patients underwent a complete clinical evaluation including recording of personal and clinical data, medical history and neurological examination. Demographic and clinical data are summarized in Table 1. In particular, we evaluated the degree of spasticity in the lower limbs using the MAS score; the severity of the disease through specific scales for each disease and including NIHSS, EDSS and ASIA; the degree of disability using the functional independence measure (FIM) and the Barthel index (BI); the presence and intensity of neuropathic pain with the neuropathic pain symptom inventory scale (NPSI) and the intensity of the overall pain sensation through a numerical rating scale (NRS, graded from 0 = no sensation to 10 = unbearable pain, with pain threshold verbally anchored to 5) [40].

### 5.3. Neurophysiological Evaluation

Neurophysiological evaluation, including sensory threshold (ST), NWR threshold (Th) and TST of the NWR, was performed on the lower limb that was affected by spasticity, or the more severely affected one in case of bilateral spasticity by using a validated method [21]. Patients were seated in a room maintained at constant temperature (23 ± 2 °C). The lower limbs were positioned to ensure the greatest muscle relaxation, compatible with the degree of spasticity, by knee flexed to 130° and ankle to 90°. Sural nerve was stimulated percutaneously by a couple of Ag/AgCl surface electrodes applied behind the lateral malleolus.

The transcutaneous electrical stimulation consisted of a train of five individual 1-ms constant current pulses delivered at 200 Hz (equal to an interstimulus interval of 5 ms) and perceived as a unique stimulus. Each train was released in a random fashion every 25–40 s (Digitimer DS7A, UK). Electromyographic reflex responses were recorded from the capitis brevis of the biceps femoris via surface electrodes (Ag/AgCl) using a bandpass filter between 3 Hz and 3 kHz (Amplifier, Mangoni Biomedica, Pisa, Italy). The analysis time was 300 ms with the sensitivity set at 100 mV. Each reflex response was rectified and integrated in the 80–130 ms post-stimulus interval (CED Powerlab interface 1401 and Signal Software, Cambridge, UK).

The ST was determined on the basis of a sequence of stimuli of increasing intensity in 1 mA steps randomly delivered every 10–20 s. Subjects were asked to indicate verbally the stimulation level at which they became aware of sensory sensations. The staircase method was used to evaluate NWR threshold (Th), defined as the intensity of stimulation able to generate a stable reflex response by an amplitude exceeding 20 µV for more than 10 ms in the 80–130 ms time window post stimulus. The area under the curve (area) of the NWR responses at Th were automatically measured and expressed as ms × mV. The area was preferred to the reflex amplitude because it represented a more reliable parameter of the spinal motoneuron reflex activation [41]. Subjects were asked on the intensity of pain attributed to each stimulus through the same NRS previously described.

To obtain the TST of the NWR, the sural nerve was stimulated using the previously described pulse train of 5 stimuli of 1 msec delivered at 200 Hz and repeated 5 times at a frequency of 2 Hz [21]. The current intensity was progressively increased from 2 mA, with steps of 1 mA, until the TST was detected. The TST of the NWR was defined as the lowest intensity able to evoke an NWR response in the fourth and fifth trace of the five-train series delivered at 2 Hz and accepted when three consecutive recordings gave the same threshold. The pain intensity related to the fifth stimulus of the five-train series delivered at 2Hz was rated by using the same 11-point NRS scale previously described for Th.

### 5.4. Botulinum Neurotoxin Type A treatment

OnabotulinumtoxinA in doses ranging from 50 units to 200 units per site were injected with electromyographic guide according to patient’s weight and the degree of spasticity in the following muscles: sSoleus, gastrocnemius medialis, tibialis posterior, flexor digitorum brevis and adductor longus.

### 5.5. Study Design

This was an open label study. After the enrollment, a first clinical and neurophysiological evaluation was performed before the OnabotulinumtoxinA treatment (T0). The same clinical and neurophysiological evaluation was carried out 30 days after OnabotulinumtoxinA injection (T1). Study procedures were performed by different physicians: Enrolment (CT), clinical and neurophysiological acquisitions (EB), OnabotulinumtoxinA injection (EA). The separation of roles was put in place to limit the impact of individual beliefs upon the neurophysiological parameters. In this way, the clinical neurophysiologist was not informed of the type and timing of the treatment the patients had undergone.

### 5.6. Statistical Methods

The size of the sample was calculated considering the modification of the TST (T1 vs. T0) in the overall population as the primary outcome. The following parameters were used: Confidence interval (two sided) 95%; power 80%; difference between groups of 2.0 mA; standard deviation of 3.0 mA. The minimum suggested sample size was 20; we decided to enroll 25 patients in order to control for drop-outs.

The Statistical Package for the Social Sciences (SPSS) for Windows, version 19.0, was used for all analyses (SPSS Inc., Chicago, IL, USA, 2010).

Mean values of demographic, clinical, neurophysiological (ST, NWR Th, Area and TST) and psychophysical variables (NRS) clustered for group of patients (stroke, MS, SCI) were entered in the statistical analysis.

One-way analysis of variance (ANOVA) was used to compare the mean values of the clinical and demographic characteristics, neurophysiological and psychophysical baseline variables between the different groups (stroke, MS, SCI). Paired t-tests were performed to compare the mean values of clinical, neurophysiological and psychophysical variables between T0 and T1.

All values were reported as means ± S.D. Pearson’s correlation was used to test correlations among electrophysiological and clinical variables.

The level of significance was set at *p* < 0.05 (two-tails) for all the tests.

## Figures and Tables

**Table 1:** Demographic and clinical characteristics of the study population at baseline (T0), expressed as mean values ± standard deviation.

		All Patients	Stroke	MS	SCI	ANOVA (F or χ^2^)	*p*-Value
Number (N)		25	10	10	5		
Age (years)		59.4 ± 12.6	66.5 ± 8.1	49.9 ± 8.8	39.5 ± 9.2	F (2,21) 15.699	0.001
Sex—Male (N - %)		14–56%	6–60%	3–30%	5 – 100%	**χ**^2^ (2) 3.745	0.154
Height (cm)		166.8 ± 5.7	167.1 ± 5.6	165.6 ± 5.6	169.0 ± 9.9	F (2,18) 0.301	0.744
Weight (Kg)		69.8 ± 11.8	73.7 ± 10.6	63.1 ± 12.9	69.5 ± 7.8	F (2,18) 1.961	0.170
Disease duration (years)		11.3 ± 11.9	4.7 ± 6.2	23.3 ± 8.6	18.4 ± 23.4	F (2,21) 0.266	0.001
Botulinum toxin dosage (units)		221.9 ± 135.1	218.0 ± 164.1	210.7 ± 73.2	290.0 ± 14.1	F (2,21) 12.100	0.769
First treatment with botulinum toxin (N - %)		13–52%	5–50%	3–30%	5–100%	**χ**^2^ (2) 0.705	0.703
Affected side (N - %)	R	13–52%	8–80%	4–40%	0–0%	**χ**^2^ (4) 4.419	0.352
	L	6–24%	1–10%	2–20%	3–60%		
	B	6–24%	1–10%	4–40%	2–40%		
FIM (20–140)		81.6 ± 28.5	83.3 ± 27.2	70.3 ± 32.3	105.0 ± 14.1	F (2,21) 1.125	0.344
BARTHEL INDEX (0–20)		11.5 ± 5.9	13.0 ± 4.8	6.8 ± 6.1	16.5 ± 2.1	F (2,20) 3.522	0.049
BDI (0–63)		8.0 ± 4.6	8.6 ± 4.5	8.5 ± 5.1	3.5 ± 2.1	F (2,20) 1.117	0.347
MMSE (0–30)		26.2 ± 3.7	25.5 ± 2.8	26.3 ± 5.4	29.5 ± 0.7	F (2,19) 1.461	0.257
NPSI (0–50)		8.6 ± 12.7	5.8 ± 11.9	13.6 ± 14.8	6.5 ± 9.2	F (2,18) 0.325	0.727
NRS (0–10)		2.5 ± 3.4	1.5 ± 2.7	3.7 ± 3.8	4.0 ± 5.7	F (2,18) 0.768	0.479
MAS–injected lower limb							
HIP (0–4)		1.5 ± 1.3	1.1 ± 0.9	2.3 ± 1.6	2.0 ± 0.0	F (2,20) 2.572	0.101
KNEE (0–4)		2.4 ± 1.1	1.8 ± 1.0	3.3 ± 0.5	3.0 ± 0.0	F (2,20) 4.833	0.019
ANKLE (0–4)		2.4 ± 0.9	2.3 ± 1.0	2.8 ± 0.9	2.0 ± 0.0	F (2,20) 1.035	0.374
OVERALL (0–16)		6.5 ± 3.3	5.1 ± 2.7	9.2 ± 3.7	7.0 ± 0.0	F (2,20) 2.366	0.120

MS = multiple sclerosis; SCI = spinal cord injury; R = right; L = left; B = bilateral; FIM = functional independence measure; BDI = Beck depression inventory; MMSE = mini mental state examination; NPSI = neuropathic pain scale inventory; NRS = numerical rating scale; MAS = modified Ashworth scale.

**Table 2:** Mean values ± standard deviation of the clinical scales of the overall studied population before (T0) and 30 days after (T1) the OnabotulinumtoxinA injection of the lower limb.

	T0	T1	Paired *t*-Test	*p*-Value
FIM (20–140)	81.6 ± 28.5	82.4 ± 32.1	(1,19) −0.237	0.815
BARTHEL (0–20)	11.5 ± 5.9	14.7 ± 11.6	(1,19) −1.386	0.182
NPSI (0–100)	8.6 ± 12.7	6.1 ± 8.9	(1,19) 1.873	0.077
NRS (0–10)	2.5 ± 3.4	2.0 ± 2.9	(1,19) 2.463	0.024
MAS–injected lower limb				
HIP (0–4)	1.5 ± 1.3	1.4 ± 1.1	(1,19) 0.900	0.379
KNEE (0–4)	2.4 ± 1.1	1.8 ± 1.2	(1,19) 2.979	0.008
ANKLE (0–4)	2.4 ± 0.9	1.9 ± 1.2	(1,19) 3.123	0.006
OVERALL (0–16)	6.5 ± 3.3	4.8 ± 3.1	(1,19) 3.478	0.003

FIM = Functional independence measure; NPSI = Neuropathic pain scale inventory; NRS = numerical rating scale; MAS = modified Ashworth scale.

**Table 3:** Mean values ± standard deviation of the neurophysiological parameters according to patient groups in the injected lower limb at baseline (T0).

	Stroke	MS	SCI	ANOVA	*p*-Value
ST (mA)	1.8 ± 1.6	1.2 ± 0.9	2.0 ± 2.3	F (2,21) 1.150	0.336
NWR Th (mA)	15.9 ± 3.2	27.5 ± 16.0	18.0 ± 16.9	F (2,19) 1.199	0.323
NRS Th	5.9 ± 1.6	6.0 ± 2.0	5.0 ± 0.4	F (2,19) 0.270	0.766
Area (msec*mA)	3516.4 ± 3272.0	1690.7 ± 909.1	8797.6 ± 10,783.0	F (2,19) 3.744	0.043
TST (mA)	6.6 ± 2.3	7.2 ± 4.5	9.6 ± 8.9	F (2,20) 0.416	0.665
NRS 5°st	5.3 ± 1.	4.3 ± 0.8	3.0	F (2,20) 1.495	0.248

MS = multiple sclerosis; SCI = spinal cord disease; ST = sensory threshold; NWR Th = nociceptive withdrawal reflex threshold; NRS Th = numerical rating scale (0–10) at NWR Th; area = area under the curve of the NWR at Th; TST = temporal summation threshold of the NWR; NRS 5°st = numerical rating scale (0–10) at 5° stimulus of the TST.

**Table 4:** Mean values ± standard deviation of the neurophysiological parameters of the injected lower limb in the overall population at baseline (T0) and 30 days after (T1) the OnabotulinumtoxinA injection.

	T0	T1	Paired *t*-Test	*p*-Value
ST (mA)	1.6 ± 1.4	2.1 ± 1.6	(1,14) −1.429	0.175
NWR Th (mA)	20.9 ± 12.2	22.7 ± 10.4	(1,14) −0.807	0.433
NRS Th	5.8 ± 1.7	6.6 ± 1.9	(1,14) −2.175	0.057
Area (msec*mV)	3490.3 ± 4312.9	2230.6 ± 2125.1	(1,14) 1.599	0.132
TST (mA)	7.2 ± 4.0	9.5 ± 5.1	(1,14) −2.655	0.019
NRS 5°st	4.7 ± 1.3	4.8 ± 1.7	(1,13) 0.16	0.856

ST = sensory threshold; NWR Th = nociceptive withdrawal reflex threshold; NRS Th = numerical rating scale (0–10) threshold; area = area under the curve of the NWR at Th; TST = temporal summation threshold of the NWR; NRS 5° st = numerical rating scale (0–10) at 5° stimulus of the TS.
